# AAV-Mediated Restoration of Dystrophin-Dp71 in the Brain of Dp71-Null Mice: Molecular, Cellular and Behavioral Outcomes

**DOI:** 10.3390/cells13080718

**Published:** 2024-04-20

**Authors:** Ophélie Vacca, Faouzi Zarrouki, Charlotte Izabelle, Mehdi Belmaati Cherkaoui, Alvaro Rendon, Deniz Dalkara, Cyrille Vaillend

**Affiliations:** 1 Université Paris-Saclay, CNRS, Institut des Neurosciences Paris-Saclay, 91400 Saclay, Francemehdi.belmaati@gmail.com (M.B.C.); 2 Department of Therapeutics, Sorbonne University, Institut de la Vision, 75012 Paris, France; alvaro.rendon@inserm.fr (A.R.);

**Keywords:** dystrophin-Dp71, glia, AAV, gene therapy, behavior, brain, central nervous system

## Abstract

A deficiency in the shortest dystrophin-gene product, Dp71, is a pivotal aggravating factor for intellectual disabilities in Duchenne muscular dystrophy (DMD). Recent advances in preclinical research have achieved some success in compensating both muscle and brain dysfunctions associated with DMD, notably using exon skipping strategies. However, this has not been studied for distal mutations in the *DMD* gene leading to Dp71 loss. In this study, we aimed to restore brain Dp71 expression in the Dp71-null transgenic mouse using an adeno-associated virus (AAV) administrated either by intracardiac injections at P4 (ICP4) or by bilateral intracerebroventricular (ICV) injections in adults. ICP4 delivery of the AAV9-Dp71 vector enabled the expression of 2 to 14% of brain Dp71, while ICV delivery enabled the overexpression of Dp71 in the hippocampus and cortex of adult mice, with anecdotal expression in the cerebellum. The restoration of Dp71 was mostly located in the glial endfeet that surround capillaries, and it was associated with partial localization of Dp71-associated proteins, α1-syntrophin and AQP4 water channels, suggesting proper restoration of a scaffold of proteins involved in blood–brain barrier function and water homeostasis. However, this did not result in significant improvements in behavioral disturbances displayed by Dp71-null mice. The potential and limitations of this AAV-mediated strategy are discussed. This proof-of-concept study identifies key molecular markers to estimate the efficiencies of Dp71 rescue strategies and opens new avenues for enhancing gene therapy targeting cognitive disorders associated with a subgroup of severely affected DMD patients.

## 1. Introduction

Duchenne muscular dystrophy (DMD) is an X-linked multifactorial handicap caused by mutations in the dystrophin (or *DMD*) gene. In addition to progressive and fatal muscle degeneration, DMD is frequently associated with a range of central comorbidities, such as intellectual disability, anxiety, social problems and neuropsychiatric disorders. These conditions further contribute to important healthcare, educational and quality-of-life concerns [1], and their treatment remains a challenging venture due to the multifactorial etiology of brain involvement in DMD. Indeed, several internal promoters in the *DMD* gene give rise to a range of full-length (Dp427) and shorter C-terminal products (Dp260, Dp140 and Dp71) with cell-specific expression in the central nervous system (CNS) [2]. Consequently, the position of the mutation within the *DMD* gene may alter the expression of distinct brain dystrophins and lead to variable profiles of cognitive and behavioral disturbances. All mutations affect the expression of the neuronal Dp427 and lead to moderate cognitive deficits, while in about one-third of patients, mutations that are more distal induce a cumulative loss of the shorter dystrophin products and more severe intellectual disabilities, with Dp71 deficiency being a pivotal aggravating factor [3,4].

Previous mouse studies have provided information on the localization of Dp71 in brain and some outcome measures that could be used to evaluate the efficacy of therapeutic strategies designed to compensate behavioral impairments associated with Dp71 deficiency. A fraction of Dp71 is expressed in neuronal synapses [5], but the majority is detected in perivascular astrocytes throughout the forebrain, as well as in radial glial cells in the retina (Müller glial cells) and cerebellum (Bergmann glial cells) [6,7,8]. Converging evidence of the medical importance of DMD underscores the critical involvement of Dp71 at the glial–vascular interface in composing the blood–brain and blood–retinal barriers (BBBs and BRBs) [9,10,11], suggesting that glial dysfunction is a core mechanism underlying cognitive dysfunction in the most severely affected DMD patients. As other dystrophins, Dp71 is a critical component of a multiprotein transmembraneous complex that bridges proteins of the extracellular matrix to cytoskeleton’s actin and signaling proteins. In both retina and brain, this Dp71-associated complex comprises dystroglycan, α1-syntrophin and α-dystrobrevin. It is expressed in the endfeet of macroglial cells that surround capillaries and form glia limitans, where it is required for the proper localization of AQP4 water and Kir4.1 potassium channels [8,12,13,14,15]. Consequently, the absence of Dp71 or α1-syntrophin in mice drastically reduces expression of AQP4, leading to alterations of astrocyte properties, central water homeostasis and potassium extracellular concentrations [6,7,16,17,18,19,20]. In line with the known role of AQP4 channels in regulating neuronal activity and synaptic plasticity, the absence of Dp71 in Dp71-null mice leads to enhanced neuronal excitation and reduced synaptic plasticity in both cortical, hippocampal and cerebellar regions. At the behavioral level, this was associated with enhanced anxiety, reduced exploration and deficits in spatial learning, spatial working memory and cognitive flexibility [5,21,22,23]. This supports key roles for Dp71 in DMD central comorbidities and the need to develop treatments targeting this specific dystrophin.

Developing genetic therapies to restore the expression of brain dystrophins is a challenging venture. New molecular tools, such as antisense-oligonucleotide-mediated exon skipping, have been developed over the last decades to restore the *DMD*-gene reading frame and rescue Dp427 expression. While these approaches hold realistic prospects of restoring muscle function in DMD [24,25], only a few studies have achieved relative success in compensating some behavioral disturbances associated with the loss of brain Dp427 [26,27,28]. Moreover, the most distal mutations affecting Dp71 are not eligible for this type of gene correction strategy, as exon skipping would suppress important functional domains of the protein normally required for key interactions with the Dp71-associated protein complex. Therefore, molecular tools and gene-therapy approaches specifically targeting Dp71 have long been lacking, precluding preclinical interventions in mouse models of DMD. However, we previously engineered an adeno-associated virus (AAV) containing the complete murine Dp71 sequence under the control of a strong ubiquitous chicken β-actin (CBA) promoter as a first tool of a Dp71 replacement strategy in CNS. AAV vectors present several advantages that are essential to developing gene therapy in the brain, as they are nonpathogenic, result in long-term expression of the encoded gene and are capable of transducing nondividing cells. Moreover, we first found that, in the retina, differences in the permeability of the inner limiting membrane of Dp71-null mice favor a larger capacity for AAV vectors to transduce retinal cells, supporting the hypothesis that Dp71 loss abnormally enhances the BRB permeability [9,29]. We then showed that the AAV-Dp71 vector has the potential to restore Dp71 expression and its localization with other molecular partners in the retina of Dp71-null mice, including with AQP4 and Kir4.1 channels [9]. More recently, we showed a full rescue of defective electroretinographic responses following treatment with this vector in Dp71-null mice, suggesting that this gene-therapy strategy has the potential to restore Dp71 function in CNS [30].

In the present study, we used the same vector construct allowing for the bicistronic expressions of Dp71 and GFP, and we selected the AAV9 capsid serotype that shows widespread transduction efficacy and capacity to cross vascular barriers in the mouse brain, which has led to applications in gene therapy trials for various neuropediatric diseases [31,32]. This AAV9-GFP-2A-Dp71 vector was delivered in two distinct groups of Dp71-null and WT littermate mice, either by intracardiac injections at postnatal day 4 (P4) (i.e., just after the postnatal peak of astrocytogenesis to favor the transduction of glial elements) or by stereotaxic bilateral intracerebroventricular injections at 6–8 weeks of age. An AAV9-GFP vector was also injected in control Dp71-null mice. We then assessed the expression levels of Dp71 in the forebrain and cerebellar regions, its cellular localization and that of α1-syntrophin and AQP4 channels, and the level of anxiety in order to evaluate the efficacy of the Dp71 rescue.

## 2. Materials and Methods

### 2.1. Animals

Dp71-null mice were generously provided by Prof. David Yaffe (Weizmann Institute of Science, Rehovot, Israel), who created this mouse line by homologous recombination, by substituting most of the first exon of Dp71 and a small portion of the first intron with the promoter-less gene coding β-Gal-neomycin resistance chimeric protein. This insertion induces a knock-in of the Dp71 expression without modifying the expressions of other dystrophins [33]. Dr. Alvaro Rendon (Institut de la Vision, Paris, France) crossed Dp71-null mice with C57BL/6JRj mice (JANVIER LABS, Le Genest-Saint-Isle, France) at CDTA (CNRS, Orléans, France) over more than 10 generations. Production in our institute consisted in crossing heterozygous females with C57BL/6JRj males, thus generating Dp71-null and wild-type littermate control males (WT). PCR analysis of tail DNA were used to determine the genotypes. Mice were kept with food and water ad libitum under a standard 12 h light–dark cycle (light on 7.00 a.m.). Mouse care and experimental procedures respected the European Communities Council Directive (CEE 86/609/EEC), EU Directive 2010/63/EU, French National Committee (87/848) and Ethic Committee (Paris Center et Sud, no. 59).

### 2.2. AAV-Vector Administration and Experimental Groups

Our group developed an AAV vector to restore Dp71 expression in the Dp71-null mouse by cloning the viral 2A peptide for bicistronic expression of GFP (green-fluorescent protein) and Dp71 genes. The GFP-2A-Dp71 fragment, synthesized by GENEWIZ Inc. (Leipzig, Germany), was cloned into a self-complementary (sc) AAV plasmid containing a ubiquitous, strong CBA promoter [34] and inverted terminal repeat regions for packaging of the sequence of interest. The term ‘CBA promoter’ is synonymous with CAG promoter [35]. The scAAV-CBA-GFP-2A-Dp71 plasmid was initially validated in the retina [9,29,30]. Control scAAV9-GFP vector only included the GFP sequence under control of CBA promoter.

As previously described, we produced the recombinant AAV vectors by plasmid cotransfection method [29]. In summary, we purified the resulting lysates via iodixanol gradient ultracentrifugation [36], concentrated and buffer exchanged the 40% iodixanol fraction using Amicon Ultra-15 Centrifugal Filter Units (Merck Millipore, Molsheim, France) and then tittered our vector stocks of DNase-resistant vector genomes by real-time PCR relative to a standard [37]. 

We injected the first groups of mice (n = 3 WT and 3 Dp71-null mice) with the control AAV9-GFP vector only to analyze the vector transduction territories in the brain one month following intracardiac injection at postnatal day 4 (ICP4). Then, two distinct cohorts of mice received one of the two vectors either by the ICP4 method (ICP4) (n = 7 Dp71-null mice with AAV9-GFP-2A-Dp71; n = 9 WT with AAV9-GFP; n = 7 Dp71-null mice with AAV9-GFP) or by stereotaxic intracerebroventricular (ICV) injections at the age of 6-8 weeks (n = 6 Dp71-null mice with AAV9-GFP-2A-Dp71; n = 6 WT with AAV9-GFP; n = 6 Dp71-null mice with AAV9-GFP). Both cohorts were submitted to a short (2.5-week duration) behavioral test battery at the age of 10 weeks for the ICP4 mice (66 days after ICP4 injection) or 10-12 weeks for the ICV mice (30 days after ICV).

For intracardiac injections, P4 pups were anesthetized by placing them for 2–3 min on an iced surface. The vector solution (20 µL) was injected rapidly with a 31-gauge syringe into the heart, and the mouse was returned to its home cage, with the rest of the progeny and the mother, to avoid hypothermia and anxiety.

We also performed intracerebroventricular (ICV) injections in 6- to 8-week-old Dp71-null and WT littermate male mice profoundly anesthetized by a single intraperitoneal injection of ketamine (95 mg/kg)/medetomidine (1 mg/kg). We bilaterally injected the AAV9-GFP-2A-Dp71 and control AAV9-GFP (1 × 10^14^ vg/mL) vectors into both lateral brain ventricles (−0.5 mm from bregma; 1 mm lateral; −2 mm from dura) [38]. We infused a volume of 5 μL at a rate of 0.3 μL/min in each ventricle. A systemic analgesic was injected postoperatively (buprenorphine, 0.05 mg/kg IP). We placed each mouse under heated conditions until it woke up and was then placed back in its home cage with its littermates.

### 2.3. Real-Time Quantitative PCR

We extracted total RNA with the NucleoSpin RNA kit (Macherey-Nagel, Düren, Germany), and we performed reverse transcription using Transcriptase inverse SuperScript™ III (Invitrogen, Carlsbad, CA, USA). We performed PCR amplification of the Dp71 cDNA using Master plus SYBR Green I (Roche Diagnostics, Risch-Rotkreuz, Switzerland) on a LightCycler instrument (Roche Products, Basel, Switzerland). We designed PCR primers using the ApE (A plasmid Editor) software (v3.1.4, 11 October 2023). For relative comparison, we analyzed the Ct values of real-time PCR data with the ΔΔCt method. We normalized the amount of cDNA to the standard internal control obtained using UBC (ubiquitin C) primers.

### 2.4. Western Blots

Following cervical dislocation, we dissected the hippocampus, cortex and cerebellum on ice, and we froze all samples in liquid nitrogen in Eppendorf with ceramic beads (MO Bio kit, Qiagen, Hilden, Germany). We homogenized the samples by centrifugation (6800× *g*, 20 s, 2–3 times) in a RIPA–sodium dodecyl sulfate (SDS) lysis buffer containing a Halt protease inhibitor cocktail (1×). We quantified proteins with the Pierce™ BCA protein assay kit (Life Technologies, Saint-Aubin, France). We separated proteins (50 μg) using NuPAGE^®^ Tris-Acetate 4–12% gradient gels (Invitrogen) at 150 V for 1 h 15, and we electrotransferred proteins (30 V, 1 h) onto polyvinylidene difluoride membranes (PVDF; Millipore, Burlington, MA, USA). We blocked the PVDF membranes for 1 h at room temperature (RT) using the Pierce™ Milk Blocking buffer (Life Technologies, Saint-Aubin, France). We then we incubated membranes overnight at 4 °C in blocking buffer with the primary polyclonal H4 antibody that recognizes all dystrophins (H4, 1:5000). We washed membranes with PBS-Tween 0.5%, and we incubated them for 1 h at RT with a goat anti-rabbit secondary antibody conjugated to horseradish peroxidase (Jackson Immunoresearch Laboratories, Montlucon, France) diluted in PBS-Tween 0.5%. We used the monoclonal anti-vinculin primary antibody, hVin-1 (1:15,000, Sigma-Aldrich, Saint-Louis, MI, USA), as an internal control to normalize the expression levels in each sample. We performed enhanced chemiluminescence (ECL) using ECL plus a Western blotting detection system (GE Healthcare, Munich, Germany), and we analyzed gels with a C-Digit Li-Cor scanner associated with the Image Studio 4.0 software.

### 2.5. Immunochemistry

We cut fresh-frozen brain hemispheres on a cryostat, and we collected 30 µm thick cryosections, which were stored at −80 °C. Prior to the immunostainings, we postfixed the slices at −20 °C for 5 min in acetone/methanol (1:1). We washed the tissue sections 3 times in 0.1 M phosphate-buffered saline (PBS). We incubated the slides for 45 min in a blocking solution composed of 10% normal goat serum, 0.3% Triton X-100 and 1% bovine serum albumin (BSA). Then, we incubated the sections overnight at 4 °C with the primary antibody diluted in the blocking buffer. The next day, we washed and then incubated them for 1 h at RT with secondary antibodies (1:500) and finally cover-slipped the sections using a medium containing DAPI (Fluoromount-G, Clinisciences, Nanterre, France). We did not observe any staining when the secondary antibody was applied alone. 

For triple immunostaining, we used the anti-Glial Fluorescent Protein polyclonal chicken antibody (anti-GFP; 1:1000, ab13970, Abcam, Cambridge, UK), anti-Glial Fibrillary Acidic Protein polyclonal rabbit antibody (anti-GFAP; 1:1000, Z0334, Dako/Agilent, Santa Clara, CA, USA) and anti-NEUronal Nuclei clone A60 monoclonal mouse antibody (anti-NeuN; 1:1000, MAB377, Sigma-Aldrich, Saint-Louis, MI, USA). For double immunostaining, we used the following primary antibodies: polyclonal rabbit anti-dystrophin targeting C-terminus (H4; exons 78–79) (1:400) and anti-β-dystroglycan (LG5; 1:400), generously provided by D. Mornet (Montpellier, France); polyclonal rabbit anti-Aquaporin 4 (AQP4-300-314; AQP-014; 1:400; Alomone labs, Jerusalem, Isreal) and anti-α1-syntrophin antibodies (APZ-021, 1:200; Alomone labs, Jerusalem, Isreal) and anti-GFAP monoclonal mouse antibody (G3893; 1:500, Sigma-Aldrich, Saint-Louis, MI, USA). The IgG H&L Alexa Fluor^®^ 488 goat anti-chicken, Alexa Fluor^®^ 546 goat anti-rabbit and Alexa Fluor^®^ 647 goat anti-mouse were used as secondary antibodies (1:400, Abcam, Cambridge, UK).

### 2.6. Confocal Microscopy

We collected images using a laser scanning confocal microscope (LSM 700; Zeiss, Rueil-Malmaison, France). For both genotypes, we took sequential dual-channel recording of multilabeled sections at equivalent locations and exposure times. We adjusted the intensity of the excitation lines for DAPI and secondary antibodies to avoid fluorochromes’ cross-excitation.

### 2.7. Behavioral Testing

General procedure: The impact of Dp71 partial rescue on behavioral parameters was analyzed following ICP4 and ICV injections in two independent cohorts. The control groups included WT and Dp71-null mice injected with AAV9-GFP vectors (WT-C and Dp71-C) and Dp71-null mice treated with the AAV9-GFP-Dp71 (Dp71-T). For both experiments, the sequence of behavioral test was similar and lasted 2.5 weeks: On the first day, mice were successively submitted to light–dark choice (morning) and elevated plus maze (afternoon) anxiety tests. Forty-eight hours later, they were submitted to a 30 min session of exploration of an open field and one week later to an object-location recognition test.

Elevated plus-maze: The maze had two closed arms (20 cm × 8 cm × 25 cm) and two open arms (20 cm × 8 cm), and it was placed on a vertical support at 65 cm above the floor. We adjusted the illumination to 150 lux in open arms and 30 lux in closed arms. We placed mice individually at the center of the maze, facing a closed arm. We manually scored the number of entries and the time spent in each type of arms in a 5 min duration.

Light–dark choice: The device had Plexiglas walls (20 cm high) and the dark compartment (15 cm × 15 cm; <15 lux) was connected by a trap door (6 cm × 6 cm) to a well-lit white compartment (40 cm × 15 cm). We provided a bright illumination by a source of light placed at the end of the white compartment, on the opposite side of the trap door, which created a lighting gradient (50 to 600 lux from door close end of the lit compartment). We placed each mouse in the dark compartment during 10 s, then we opened the trap door giving access to the lit compartment during 5 min. We manually scored the latency, number of entries and total time spent in the lit compartment.

### 2.8. Statistics

The data are expressed as the means ± standard error of the mean (SEM). Genotype and treatment effects were analyzed using the nonparametric Mann–Whitney test. We considered that *p*-values < 0.05 were statistically significant. The normal approximation was used to calculate the significance of the U in the Mann–Whitney test when comparing behavioral parameters in the ANY-maze [39].

## 3. Results

### 3.1. Rescue of Dp71 and Associated Proteins following Neonatal Intracardiac Injection

**Figure 1:**
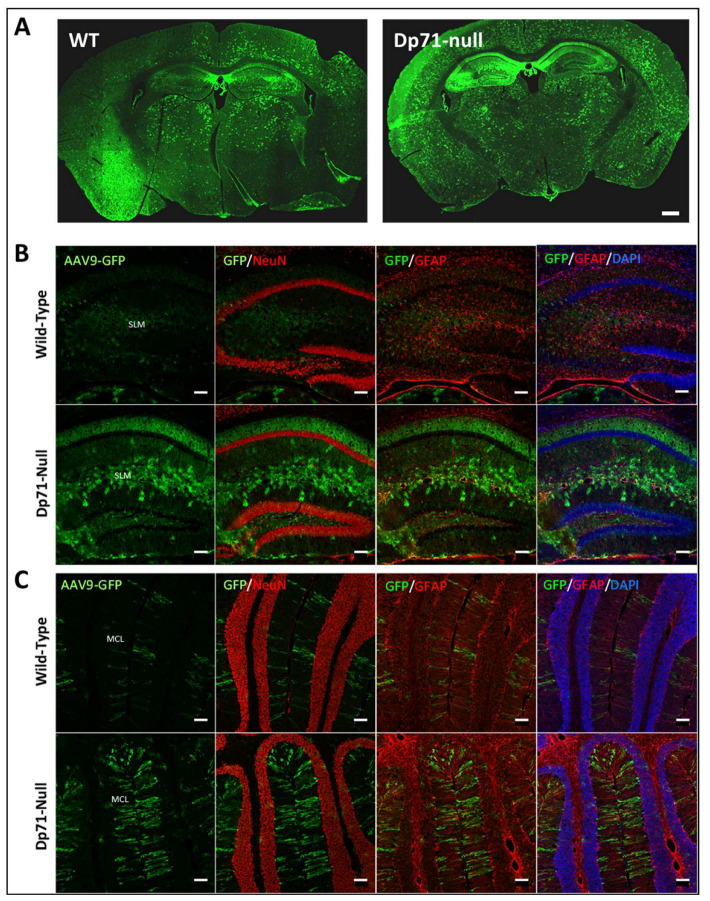
Transduction territories of the AAV9-CAG-GFP vector following intracardiac administration: (**A**) GFP expression (green) in whole-brain sections of wild-type (left) and Dp71-null (right) mice after intracardiac injection at postnatal day 4 (ICP4) of AAV9-GFP vector (scale bar: 500 µm); (**B**,**C**) colocalization of GFP expression (green) and the neuronal marker NeuN (red) or astrocyte marker GFAP (red) in the hippocampus (**B**) and cerebellum (**C**). The nuclear marker, DAPI, is shown in blue (scale bar: 100 µm). Note that AAV9 intracardially injected at P4 widely transduced non-neuronal, mostly glial, cells in both wild-type and Dp71-null mice. SLM: *stratum lacunosum moleculare*; MCL: molecular cell layer.

Four weeks after intracardiac injections of 3 WT and 3 Dp71-null mice with a control AAV9-CBA-GFP vector at postnatal day 4, we observed widespread GFP expression in the whole brain in both genotypes (Figure 1A), thus demonstrating the efficiency of AAV9 to transduce brain tissues with this delivery method. The colabeling of GFP with NeuN to visualize neurons, as well as with GFAP to identify astrocytes, revealed a lack of GFP signals in the neuronal layers of both the hippocampus and cerebellum (Figure 1B,C). In Dp71-null mice, GFP was largely expressed in other layers of the hippocampus, with strong signal in astrocyte-like elements in the stratum lacunosum moleculare (SLM), as well as in the radial processes of Bergmann glial cells in the molecular cell layer (MCL) of the cerebellum. GFP was partly colocalized with anti-GFAP immunofluorescence, suggesting its expression in a subset of glial elements. A weaker signal was observed in tissue sections from WT mice, suggesting better transduction of AAV vectors in the absence of Dp71, as reported in retinal studies [29]. 

**Figure 2:**
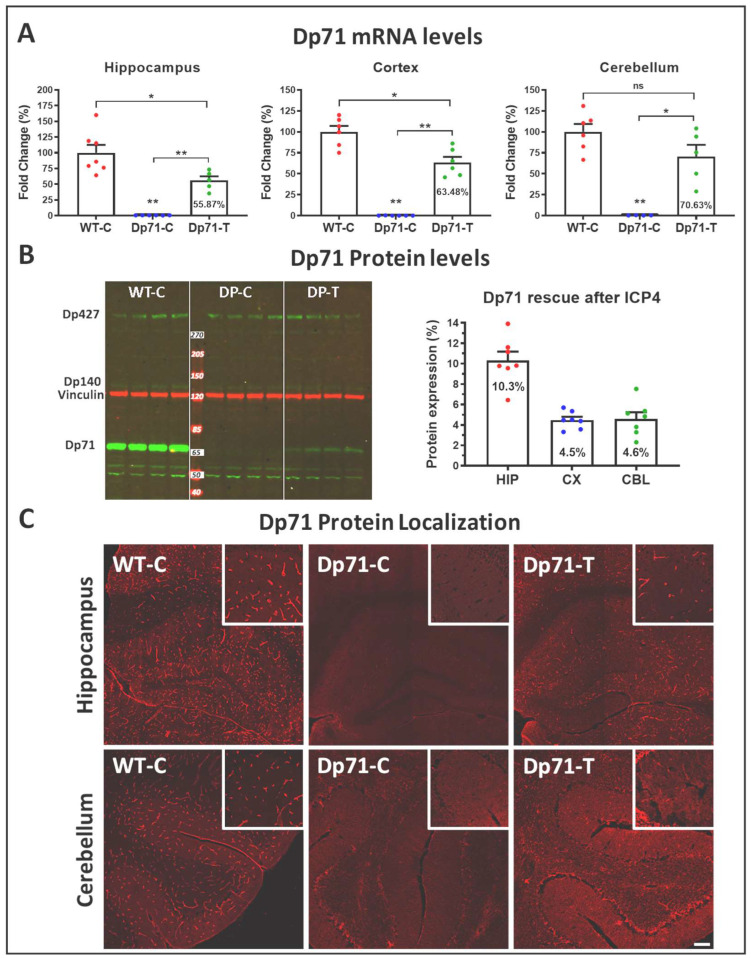
Dp71 re-expression following intracardiac injections at postnatal day 4 (ICP4). Mice were injected with the AAV9-CAG-GFP control vector (WT-C, Dp71-C) and AAV9-CBA-GFP-2A-Dp71 vector (Dp71-T). (**A**) Dp71 mRNA levels quantified by qPCR in the three groups of mice in the hippocampus, cortex and cerebellum, as indicated. (**B**) Dp71 protein levels quantified using Western blots in the three groups of mice in the hippocampus (HIP), cortex CX) and cerebellum (CBL). The image on the left shows an example of a Western blot with 4 mice from each group and the detection using the H4 antibody of dystrophins Dp427, Dp140 and Dp71 (green). Vinculin (red) was used as control protein for normalization. Importantly, note that all (n = 7) treated mice showed Dp71 protein rescue. (**C**) Dp71 localization by immunofluorescence staining using the pan-specific H4 antibody (red) on 12 µm cryosections. Images were taken at ×10 and ×0.5 magnifications (inserts: ×20 and ×1 magnifications) (scale bar: 100 µm). * *p* < 0.05, ** *p*< 0.01, ns for non-significant, Mann–Whitney test.

Following these encouraging results, we injected a cohort of 9 WT and 7 Dp71-null mice with the AAV9-CBA-GFP vector to constitute control groups, and 7 Dp71-null mice with the AAV9-CBA-GFP-2A-Dp71 vector to evaluate its potential to re-express Dp71 in these tissues. Nine weeks after the AAV vector injections, we harvested the brain to perform mRNA and protein extractions, and we kept one hemisphere of each individual for cryosections. The Dp71 mRNA levels were quantified by qPCR and expressed relative to the WT levels, showing 56% expression in the hippocampus, 63.5% in the cortex and 71% in the cerebellum, which suggest a large and relatively homogeneous diffusion of the AAV9 serotype in the whole brain (Figure 2A). However, Dp71 protein quantification by Western blot revealed a lower restoration of only 10.3% in the hippocampus and even lower in the cortex (4.5%) and cerebellum (4.6%) (Figure 2B). Brain cryosections labeled with a pan-specific antidystrophin antibody (H4) were then analyzed (Figure 2C). We observed the typical labeling of Dp71 protein along the walls of blood capillaries in the WT controls (WT-C). This was also observed in the treated Dp71-null mice (Dp71-T) (i.e., mice injected with the AAV9-CBA-GFP-2A-Dp71 vector), yet to a much lower extent, showing that the partially rescued Dp71 protein was appropriately relocalized in perivascular glial cell endfeet. A larger number of vessels were labeled with the anti-dystrophin antibody in the hippocampus than in the cerebellum of Dp71-T mice, which confirm the differences among structures revealed by the Western blot quantifications.

**Figure 3:**
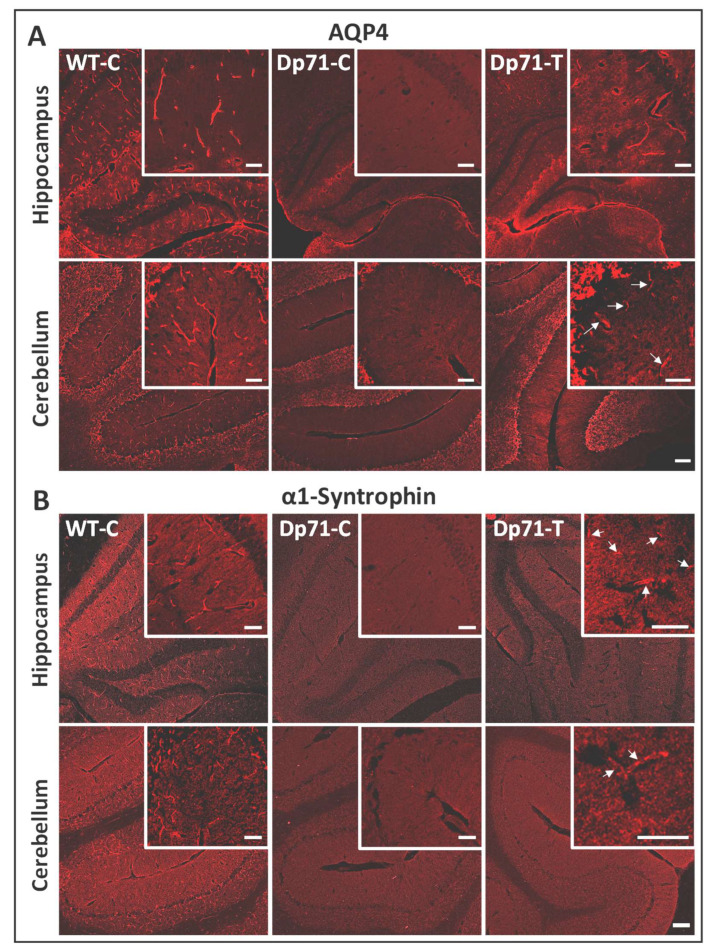
Expressions of AQP4 and α1-syntrophin along the walls of blood vessels after intracardiac administration (ICP4) of the AAV vectors. (**A**,**B**) For both antibodies, the immunofluorescent (IF) staining is shown in tissue sections of the hippocampus and cerebellum, as indicated, in Dp71-null mice injected with the AAV9-CBA-GFP-2A-Dp71 vector (Dp71-T) and with the control vector (Dp71-C, WT, C). (**A**) Immunostaining of AQP4 (red) on 12 µm cryosections. Images taken at ×10 and ×0.5 magnifications (inserts: ×20 and ×1 magnifications). The white arrows indicate labeled vessels (scale bar: 100 µm). (**B**) Immunostaining of α1-syntrophin (red) on 12 µm cryosections. Images were taken at ×10 and ×0.5 magnifications (inserts: ×20 and ×1 magnifications). The white arrows indicate labeled vessels (scale bar: 100 µm).

We then performed immunostaining of two dystrophin-associated proteins (DAPs) known as components of the Dp71-associated protein complex, as follows: AQP4 water channel and α1-syntrophin (Figure 3). They were both localized around blood vessels in sections from WT-C. In contrast, they were both strongly downregulated in absence of Dp71, as shown in sections from Dp71-null mice injected with the control AAV vector (Dp71-C). In treated Dp71-null mice (Dp71-T), we observed very clearly and intensively the localization of AQP4 around the capillaries in the hippocampus (Figure 3A). In the cerebellum, there was a weaker expression of AQP4, only in some capillaries, in accordance to the weaker rescue of Dp71 protein in this region. For α1-syntrophin, the restoration was weak and only visible around a few capillaries in both the hippocampus and cerebellum (Figure 3B).

### 3.2. Rescue of Dp71 and Associated Proteins following Intracerebroventricular Injection in Adults

**Figure 4:**
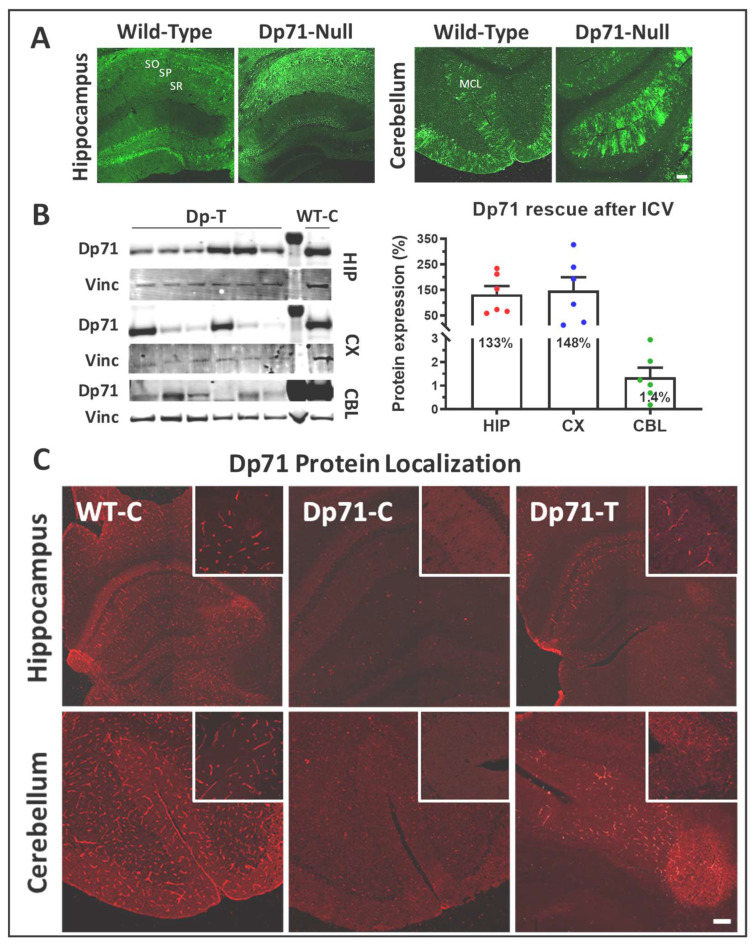
Dp71 re-expression following intracerebroventricular injections (ICVs). Mice were injected at 7 weeks with the AAV9-CAG-GFP control vector (WT-C, Dp71-C) and with the AAV9-CBA-GFP-2A-Dp71 vector (Dp71-T). (**A**) Transduction territories of the AAV9-CAG-GFP vector in the WT and Dp71-null mouse brain sections, as revealed by GFP immunofluorescent staining (green) (scale bar: 100 µm). (**B**) Dp71 protein levels in the hippocampus (HIP), cortex (CX) and cerebellum (CBL) quantified by Western blots. The image on the left shows the Western blot for the 6 treated mice (DP-T) and one WT control mice (WT-C). Dp71 was detected using the H4 antibody. Vinculin (Vinc) was used as the control protein for normalization. Importantly, note that all (n = 6) treated mice showed Dp71 protein rescue. (**C**) Dp71 expression revealed by immunofluorescent staining with the pan-specific H4 antibody (red) on 12 µm cryosections of hippocampus and cerebellum. Images were taken at ×10 and ×0.5 magnifications (inserts: ×20 and ×1 magnifications) (scale bars: 100 µm). SO: stratum oriens; SP: *stratum pyramidale*; SR: *stratum radiatum*; MCL: molecular cell layer.

In an attempt to improve the amount of Dp71 restoration in brain, we also evaluated the benefits of another delivery route based on the bilateral injection of the AAV vectors into the cerebrospinal fluid of the brain ventricles at adult age (6–8 weeks). As shown in Figure 4A, the AAV9-CBA-GFP vector showed transduction patterns that were slightly different between the WT and Dp71-null mice in the hippocampus. In WT mice, the granule cell layer of the dentate gyrus showed an intense GFP-positive signal, while a subset of neurons was labeled in the CA1 pyramidal layer. In the Dp71-null mice, we observed an intense and filamentous GFP expression extended to all layers of CA1 (strata oriens (SO), pyramidale (SP) and radiatum (SR)), as well as in the inferior suprapyramidal blade of dentate gyrus granule cell layer. 

These GFP-positive elements in Dp71-null mice did not show a typical neuronal pattern, and the GFP signal was absent in the neuronal layer of the dentate gyrus. The filamentous GFP expression likely corresponded to presumed glial elements. In the cerebellum of both genotypes, the GFP labeling was mostly detected in the typical radial processes of Bergmann glial cells in the MCL.

Nine weeks after the bilateral intracerebroventricular administration of the AAV9-CBA-GFP-2A-Dp71 vector in the Dp71-null mice, quantification of the Western blots for the dissected brain samples revealed overexpressions of the Dp71 protein in the hippocampus (133% of WT levels) and cortex (148%), while Dp71 rescue was minimal in the cerebellum (1.4%) (Figure 4B). Thus, this alternative delivery route clearly improved Dp71 restoration in the hippocampus and cortex, but it was not efficient in the rescue of Dp71 in the cerebellum.

**Figure 5:**
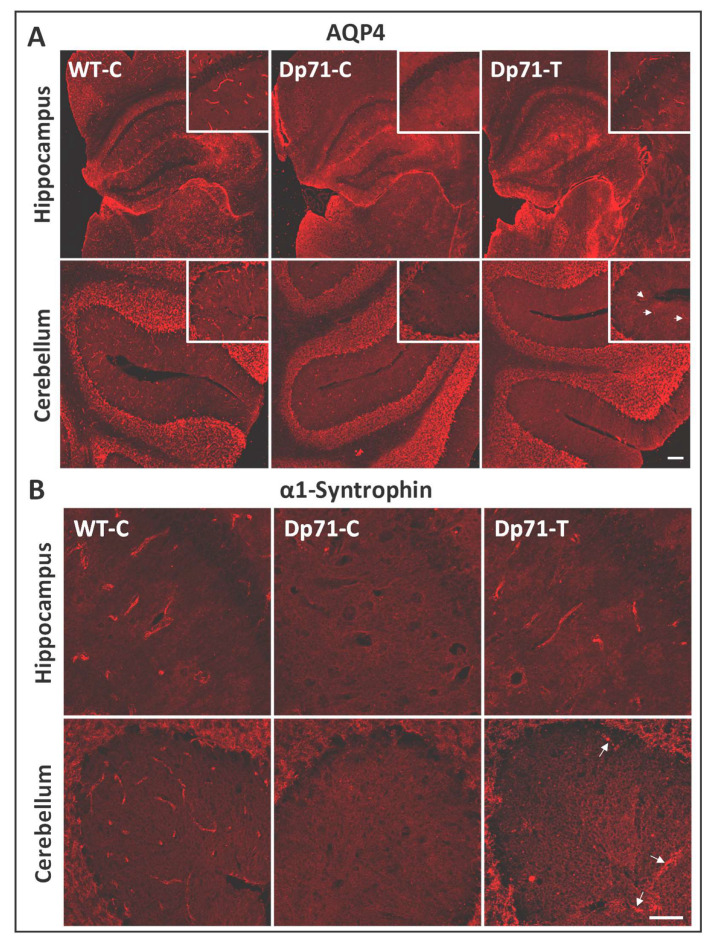
Expressions of AQP4 and α1-syntrophin along the walls of blood vessels after intracerebroventricular administration (ICV) of the AAV vectors. (**A**,**B**) For both antibodies the immunofluorescent (IF) staining is shown in tissue sections of the hippocampus and cerebellum, as indicated, in the Dp71-null mice injected with the AAV9-CBA-GFP-2A-Dp71 vector (Dp71-T) and with the control vector (Dp71-C, WT, C). (**A**) Immunostaining of AQP4 (red) on 12 µm cryosections. The white arrows indicate labeled vessels. Images were taken at ×10 and ×0.5 magnifications (inserts: ×20 and ×1 magnifications) (scale bar: 100 µm). (**B**) Immunostaining of α-1-syntrophin (red) on 12 µm cryosections. The white arrows indicate labeled vessels. Images were taken at ×20 and ×1 magnifications (scale bar: 50 µm).

Immunostaining with the pan-specific anti-dystrophin H4 antibody enabled the localization of Dp71 expression along the walls of capillaries throughout the hippocampus for all injected animals, as well as in some cerebellar sections (Figure 4C). We also performed the immunostaining of the Dp71 partners, AQP4 and α1-syntrophin (Figure 5). There was clear localization of AQP4 around the capillaries in the hippocampus and a more discreet localization in the cerebellum (Figure 5A). Moreover, we also observed suitable rescue of α1-syntrophin in the hippocampus, which was also more discreet in the cerebellum.

### 3.3. Impact of Dp71 Restoration on Behavioral Disturbances

#### 3.3.1. Intracardiac Treatment at Postnatal Day 4 (P4)

The impact of Dp71 partial rescue was first analyzed following early (P4) intracardiac (ICP4) administration of two AAV vectors defining the following three groups: one group of Dp71-null mice treated with AAV9-GFP-Dp71 vector (Dp71-T, n = 7) and two control groups composed of Dp71-null mice and WT littermate mice injected with the AAV9-GFP vector (WT-C, n = 9; Dp71-C, n = 7).

**Figure 6:**
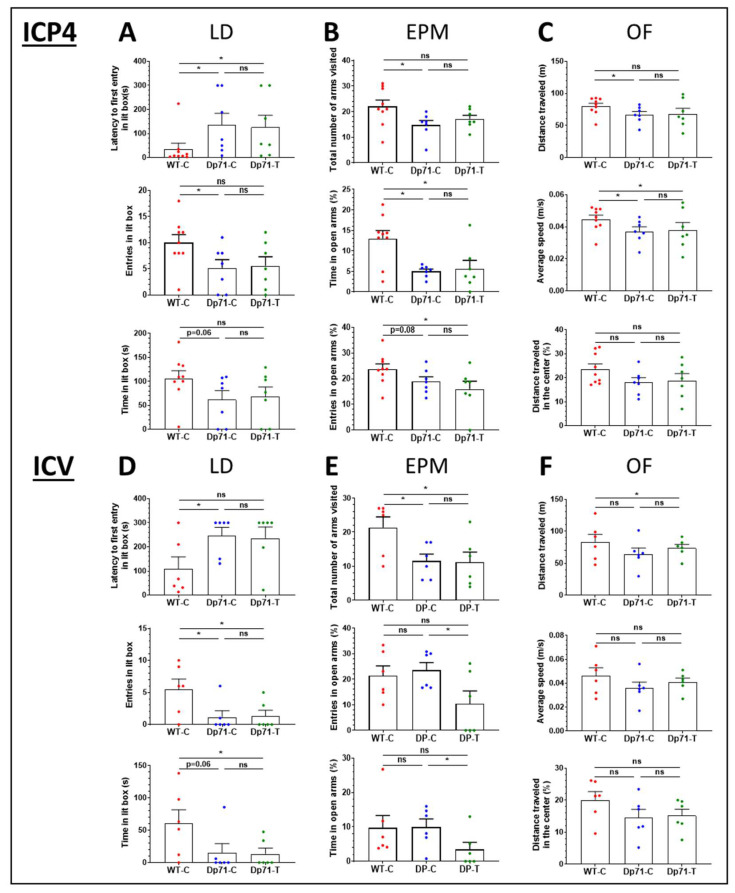
Behavioral study following ICP4 and ICV administration of the AAV vectors. (**A**–**C**) The plots show the data recorded following intracardiac injections at P4 (ICP4) in the three groups of mice (Dp71-T, n = 7; WT-C, n = 9; Dp71-C, n = 7). (**A**) Latency to the first entry in the lit box (s), number of entries and time spent in the lit box (s) in the light–dark choice test (LD). (**B**) Total number of arms visited, time spent (%) and number of entries (%) in the open arms in the elevated plus maze (EPM). (**C**) Distance traveled (m), average speed (m/s) and percent distance traveled in center in the 30 min open field exploration test (OF). (**D**–**F**) The plots show the data recorded following stereotaxic intracerebroventricular (ICV) injections at 6–8 weeks in the three groups of mice (Dp71-T, n = 6; WT-C, n = 6; Dp71-C, n = 6). (**D**) Latency to the first entry in the lit box (s), number of entries and time spent in the lit box (s) in the light–dark choice test (LD). (**E**) Total number of arms visited, time spent (%) and number of entries (%) in the open arms in the elevated plus maze (EPM). (**F**) Distance traveled (m), average speed (m/s) and percent distance traveled in center in the 30 min open field exploration test (OF). The results are the means ± SEM, * *p* < 0.05, ns for non-significant, Mann–Whitney test.

In the light–dark choice anxiety test (Figure 6A), Dp71-C mice showed longer latencies to enter the lit box (U = 9, Z = 2.38, *p* = 0.0172) and made fewer entries into this area (U = 12, Z = 2.0983, *p* = 0.0359) compared with the WT-C mice. Their time spent in the lit box was also marginally reduced (U = 14, Z = 1.853, *p* = 0.06). In addition, the two genotypes showed comparable scores for the number of hesitations to enter the lit box (U = 25.5, Z = 0.6384, *p* > 0.1) and for vertical activity (number of rearings) in the lit box (U = 18, Z = 1.437, *p* > 0.1) (Supplementary Figure S1A). The results of this test suggest a moderate increase in anxiety in the Dp71-null mice. However, comparing the behavioral responses of the Dp71-null mice treated with the AAV9-GFP-Dp71 vector with those of the control Dp71-C mice did not reveal any effect of the treatment (latency to enter lit box: U = 22, Z = 0.32, *p* = 0.74; entries in lit box: U = 22.5, Z = 0.258, *p* = 0.79; time in lit box: U = 21, Z = 0.44, *p* = 0.65). Compared with the WT-C mice, the latency to enter the lit box was still significantly longer in the Dp71-T mice (U = 12, Z = 2.06, *p* = 0.03), but the differences were no more significant for the number of entries (*p* = 0.08) and time spent in the lit box (*p* = 0.12).

In the elevated plus maze test (Figure 6B), the total number of arms visited (i.e., global exploratory activity) was reduced in the Dp71-C mice compared with the WT-C mice (U = 11, Z = 2.17, *p* = 0.029). Moreover, the percent time spent in open arms was significantly reduced in the Dp71-C mice compared with the WT-C mice (U = 10.5, Z = 2.22, *p* = 0.026), and the percent number of entries in open arms was marginally reduced (U = 15, Z = 1.75, *p* = 0.08). This also supports the presence of enhanced anxiety-related behavior in the Dp71-null mice. When the central area was also considered as an open area (Supplementary Figure S1B), both parameters showed significant reductions between these two groups (% entries: U = 10, Z = 2.224, *p* = 0.0261; % time: U = 11, Z = 2.17, *p* = 0.03). In this test again, comparing the behavioral responses of Dp71-T mice with those of control Dp71-C mice did not reveal any effect of the treatment (*p* > 0.27 for all parameters). The Dp71-T mice still showed enhanced anxiety-related behavior compared with the WT-C mice. Indeed, the Dp71-T mice still showed reductions in the percent number of entries (U = 12, Z = 2.06, *p* = 0.038) and time spent in open arms (*p* = 0.05), even if the central area was considered as an open area (% entries: U = 10, Z = 2.27, *p* = 0.022, % time: U = 10, Z = 2.27, *p* = 0.022). However, the global exploratory activity was comparable between the Dp71-T and WT-C mice (U = 17.5, Z = 1.48, *p* = 0.13) but also not different compared with Dp71-C (U = 17.5, Z = 0.9, *p* = 0.36).

During the 30 min exploration of the open field (Figure 6C), the Dp71-C mice showed reductions in distance traveled (66.81 ± 4.93 m; U = 12, Z = 2.06, *p* = 0.039) and average speed (0.037 ± 0.0028 m.s^−1^; U = 13, Z = 1.96, *p* = 0.04) compared with the WT-C (Distance: 80.27 ± 4.44; Average speed: 0.044 ± 0.0025), but they exhibited comparable maximum speed (U = 27, Z = 0.47, *p* = 0.63; Supplementary Figure S1C) and time spent immobile (WT-C: 371.01 ± 43.91; Dp71-C: 448.73 ± 65.31; U = 22, Z = 1.0056, *p* = 0.31). The percent distance traveled in the central zone was comparable between the two genotypes (U = 17, Z = 1.53, *p* = 0.12; Figure 1C), as well as the percent time spent in this area (Supplementary Figure S1C). No effect of treatment was detected by comparing these parameters between the Dp71-T mice and the Dp71-C controls (all *p* > 0.17). The parameters were also analyzed during the first 5 min, as this initial period may more strikingly reflect anxiety-related behaviors. During this period, the maximum speed was lower for the Dp71-C mice compared with the WT-C mice (U = 9, Z = 2.38, *p* = 0.017), which was due to a lower speed in the central zone (*p* = 0.029) but not in the periphery (*p* = 0.18). The percent distance run in the central zone was lower in the Dp71-C mice (U = 13, Z = 1.95, *p* = 0.05). No effect of the treatment was detected by comparing these parameters between the Dp71-T mice and Dp71-C controls.

#### 3.3.2. Intracerebroventricular Treatment in Adult Mice

In a second experiment, the impact of the Dp71 partial rescue was analyzed following intracerebroventricular administration of the AAV vectors (Dp71-T, n = 6; WT-C, n = 6; Dp71-C, n = 6).

In the light–dark choice anxiety test (Figure 6D), the Dp71-C mice showed longer latencies to enter the lit box (U = 6, Z = 1.99, *p* = 0.046), made fewer entries into this area (U = 6, Z = 2.00, *p* = 0.0447), and tended to spend less time in the lit box (U = 7, Z = 1.82, *p* = 0.06) compared with the WT-C mice. Moreover, the two genotypes showed comparable scores for the number of hesitations to enter the lit box (U = 9.5, Z = 1.37, *p* > 0.1) and for vertical activity (number of rearings) in the lit box (U = 9.5, Z = 1.52, *p* > 0.1) (Supplementary Figure S1D). The Dp71-C mice, therefore, showed the same behavioral profile in this test as in the previous experiment. Likewise, comparing the behavior of the Dp71-T mice with that of the control Dp71-C mice did not reveal any effect of the treatment (latency, number of entries and time spent in lit box: *p* > 0.9). Compared with the WT-C mice, the Dp71-T mice still showed reductions in the number of entries (U = 6, Z = 1.99, *p* = 0.045) and time spent in the lit box (U = 6, Z = 1.99, *p* = 0.046). The latency to enter the lit box was still longer in the Dp71-T mice, but this was not significant (U = 9, Z = 1.49, *p* = 0.13).

In the elevated plus maze test (Figure 6E), the total number of arms visited (i.e., global exploratory activity) was reduced in the Dp71-C mice compared with the WT-C mice (U = 6, Z = 1.93, *p* = 0.05). The percent number of entries and percent time spent in open arms was not significantly different between the Dp71-C and WT-C mice (Z < 0.65, *p* > 0.52), even when the central area was considered as an open area (Supplementary Figure S1E). Therefore, anxiety-related responses of Dp71-null mice were not readily detected in this test of this second experiment. Comparing the behavioral responses of the Dp71-T mice with those of the control Dp71-C mice revealed significant reductions in the percent number of entries (U = 6, Z = 1.93, *p* = 0.05) and percent time spent in the open arms (U = 5, Z = 2.09, *p* = 0.036). This was also observed in comparison with the WT-C mice, yet this did not reach significance (*p* > 0.1), and the total number of arms visited was still significantly lower in the treated Dp71-null mice compared with the WT-C (*p* = 0.04). 

During the 30 min exploration period in the open field (Figure 6F), the Dp71-C and WT-C mice showed statistically comparable distances traveled (WT-C: 83.20 ± 11.98 m; Dp71-C: 64.53 ± 9.3 m; U = 13, Z = 0.8, *p* = 0.42), average speeds (WT-C: 0.046 ± 0.0066 m.s^−1^; Dp71-C: 0.035 ± 0.0051 m.s^−1^; U = 13, Z = 0.8, *p* = 0.42), maximum speeds (U = 12, Z = 0.96, *p* = 0.26; Supplementary Figure S1F) and time spent immobile (WT-C: 383.62 ± 67.29 s; Dp71-C: 605.83 ± 120.01 s; U = 8, Z = 1.6, *p* = 0.10). The percent distance travelled in the central zone (U = 10, Z = 1.28, *p* = 0.2; Figure 6F), as well as the percent time spent in this zone (U = 11, Z = 1.12, *p* = 0.26; Supplementary Figure S1F), were also comparable between the Dp71-C and WT-C mice. The parameters were also analyzed for the first 5 min, as this initial period may more strikingly reflect anxiety-related behaviors. Indeed, during the first 5 min, the percent time spent in the central zone (U = 5, Z = 2.08, *p* = 0.03), as well as the distance travelled in this zone (U = 7, Z = 1.76, *p* = 0.07), was reduced in the Dp71-C mice compared with the WT-C mice. However, no effect of the treatment was detected by comparing these two parameters between the Dp71-T mice and Dp71-C controls (both *p* > 0.4). The maximum speed (U = 5, Z = 2.08, *p* = 0.037) and the percent distance in center zone travelled by the Dp71-T mice were significantly lower than those of the WT-C mice during these 5 min of the test (U = 5, Z = 2.08, *p* = 0.037).

## 4. Discussion

In this study, we tested the potential of an AAV9-mediated gene therapy to establish Dp71 expression in the brain of Dp71-null mice, either by systemic (intracardiac) injection in newborn mice or by intracerebroventricular injection (ICV) in young adults. Despite the variability in the tropism of the AAV9 vectors, depending on the genotype and delivery route, both approaches enabled the rescue and proper localization of Dp71 and proteins of the Dp71-associated complex in glial elements, yet the rescue was partial and insufficient to compensate the emotional disturbances displayed by Dp71-null mice.

Individual AAV serotypes show strong differences in their efficiency to drive gene expression in the mouse brain [40]. We chose the AAV9 capsid for its well-known tropism for the CNS. Indeed, delivery of AAV9 through the bloodstream leads to widespread transduction of brain cells in both neonatal and adult mice [41,42,43]. In neonates, AAV9 primarily transduces neurons, then astrocytes and microglia. In adult brain, astrocytes and blood vessels, are mostly transduced, with minimal transduction of neurons [41,42]. This discrepancy depending on the age of delivery seems to be linked to the progressive increase in astrocyte volume during astrogenesis, starting around E18, peaking at P3, and lasting at least until P7 [44]. Astrogenesis causes the extracellular space to shrink, giving AAV9 a lower probability of finding its receptor on neurons than on astrocytes or endothelial cells of blood vessels. Our present data show that injecting the AAV9 capsid carrying GFP expression intracardially into the bloodstream at P4 (ICP4 delivery) already benefit from this favorable configuration. Indeed, ICP4 delivery enabled efficient transduction of the whole brain and preferentially targeted glial elements in both WT and Dp71-null mice. The same vector administered to 7-week-old mice by intracerebroventricular injections (i.e., ICV delivery) very efficiently transduced predominantly glial cells in the cerebellum and hippocampus. This was characterized by a clear labeling of Bergman-cell radial extensions in cerebellum and astrocyte processes in hippocampus. A few GFP-positive neuronal cell subtypes were also detected in hippocampus, which likely corresponded to some granule cells in the dentate gyrus and a subpopulation of pyramidal neurons or putative interneurons in the CA1 cell layer. Overall, both delivery routes enabled the transduction of large brain territories, mainly in glial elements. These results were also likely favored by the specific AAV construct selected in this study. Indeed, we chose the CBA promoter that is known to drive an unprecedented level of astrocyte transduction in the CNS when used in AAV vectors [45]. Moreover, double-stranded genome conformation in self-complementary (sc) AAV9 vectors is associated with a faster rate of gene transcription onset, unlike conventional recombinant AAVs [46]. Interestingly, in both conditions we observed that the Dp71-null mice had a much greater GFP expression in glial elements compared with WT mice. This may be attributed to changes in the AAV transduction properties depending on the blood–brain barrier permeability and composition of the extracellular matrix [47], since both are modified in the absence of Dp71, as previously shown in the retina of Dp71-null mice [29].

Importantly, our comparative study revealed that the level of Dp71 rescue was different depending on the delivery protocol. Nine weeks after treating the Dp71-null mice by ICP4 with the AAV9-CBA-GFP-2A-Dp71 vector, Dp71 mRNA expressions were relatively high and homogeneous in the hippocampus (56% of WT level), cortex (64%) and cerebellum (71%). At the protein level, however, the Dp71 protein production was much lower at 10.3% in the hippocampus, 4.5% in the cortex and 4.6% in the cerebellum. The cause of this discrepancy between the mRNA and protein levels is not known, but it may depend on a lower probability for Dp71 to bind partners such as β-dystroglycan and α-syntrophin, which are normally required to anchor the Dp71-associated protein complex to the membrane. Part of the Dp71 proteins produced by the vectors might consequently be degraded because of protein instability, thus explaining the difference between the restored mRNA and protein levels. In marked contrast, using ICV injections at adult age, we achieved overexpressions of the Dp71 protein in the hippocampus (133% of WT) and cortex (148%), while a poor level of expression was quantified in the cerebellum (1.4%). Thus, Dp71 restoration was weak but relatively homogeneous among brain regions for ICP4 delivery, while overexpressions in the hippocampus and cortex, against a very weak restoration in the cerebellum, were achieved with ICV injections in adults.

We also analyzed the rescue and localization of Dp71 and key associated proteins (α1-syntrophin and AQP4 water channels) using immunofluorescence methods in tissue sections. As expected, we first showed that Dp71-associated proteins are all expressed in astrocyte endfeet around capillaries in WT mice and drastically downregulated in absence of Dp71 [8]. Following treatment of Dp71-null mice with the AAV9-Dp71 vector, we clearly detected the rescued Dp71 protein along the walls of capillaries in the hippocampus, cortex and cerebellum, which is expected if Dp71 relocalizes in glial-cell endfeet. A putative expression in synapses could not be determined, because the H4 pan-dystrophin antibody could also bind to the epitope of the full-length Dp427 dystrophin also expressed in synapses. Around capillaries, however, Dp71 rescue was heterogeneous but clearly associated with restored expressions of both α1-syntrophin and AQP4, indicating a proper localization of the Dp71-associated complex in glial endfeet. Following the ICV treatment, the relocalization of α1-syntrophin and AQP4 appeared to be larger in the hippocampus than in the cerebellum, which is consistent with the lower rescue of Dp71 in this region according to our Western blot quantifications. In all, this constitutes a proof-of-concept that AAV-mediated ectopic expression of Dp71 can restore the Dp71-associated complex in brain astrocytes. The relocalization of α1-syntrophin and AQP4 can, therefore, be viewed as a molecular outcome measure to assess the effectiveness of Dp71 rescue at the cellular level.

To further evaluate the functional consequences of Dp71 rescue, we tested the effects of these treatments on mouse emotional responses. Anxiety is a phenotype that is commonly expressed by several DMD mouse models lacking distinct dystrophins including Dp71, such as in *mdx3cv*, *mdx* and *mdx52* mice [21,48], and recent results from our laboratory indicate that enhanced anxiety is also a feature of the behavioral profile of Dp71-null mice (unpublished). Moreover, altered emotional reactivity appears to be a relevant translational phenotype also detected in DMD patients [1,49,50]. Therefore, we, here, compared the anxiety-related behavioral responses of Dp71-null mice injected with the AAV9-Dp71 vector to those of Dp71-null and WT littermates injected with the control AAV-GFP vector. First, we confirmed the presence of enhanced anxiety-related behaviors in the Dp71-null mice compared with the WT mice in the light–dark choice, elevated plus-maze and open-field tests. However, comparing the behavioral responses of the Dp71-null mice treated with the AAV9-Dp71 vector with those of the Dp71-null mice injected with the control vector did not reveal any significant effect of the treatment in any of the tests, regardless of the delivery method.

Several hypotheses could be considered in future studies to understand this lack of therapeutic effect at the behavioral level:

First, the behavioral deficits might be caused by synaptic dysfunction, and restoration of Dp71 and associated proteins in glial elements would, therefore, be inefficient in the improvement of emotional reactivity [5]. In line with this hypothesis, our AAV vector containing the full Dp71 coding sequence (without introns) only enabled the restoration of the expression of the full-length Dp71d isoform (splicing events could not occur), mostly in glial cells. Previous works have shown that the overexpression of Dp71 in retinal Müller glial cells fully normalizes the altered retinal physiology in Dp71-null mice [30]. However, Dp71 is not expressed in retinal synapses, and the rescued phenotype was, therefore, selectively related to glial dysfunction in the retina. However, in the brain, Dp71 is expressed in both glial and neuronal-synapse domains. Several Dp71 isoforms produced by alternative splicing are expressed in the mouse brain [51], and we previously showed that Dp71f is the main isoform expressed in the synapses of cultured neurons [5].

Second, the level of Dp71 re-expression and/or its distribution across brain regions might be under the threshold needed to compensate behavioral deficits. Using ICP4 delivery, Dp71 expression levels were below 15% in all regions. We previously showed that rescue of less than 30% of another brain dystrophin (Dp427) could partly improve brain and behavioral functions in other DMD mouse models, but this might be insufficient here for Dp71 rescue in Dp71-null mice [26,27,28]. With ICV delivery, however, Dp71 was overexpressed in the hippocampus and cortex, while it only reached 1.4% in the cerebellum. Although anxiety is mostly dependent on a so-called fear circuit including limbic structures such as the hippocampus and amygdala, this result suggests the possibility that some altered cerebellar processes contribute to emotional disturbances in Dp71-null mice. Indeed, the role of the cerebellum in emotional processes has largely been understudied, but recent imaging research findings in humans show evidence of hyperactivity both in the cerebellum and amygdala in social anxiety disorder [52,53], higher cerebellar baseline activity in panic disorder [54] and increased cerebellar activity in post-traumatic stress disorder [55]. Recently, anatomical tracing of mouse brain revealed hierarchical projections from the cerebellum to the social brain network, including amygdalar connections [56].

Several alternative strategies can be considered in future studies to address these hypotheses. Distinct promoters and AAV serotypes should be considered to favor the rescue of Dp71 in neuronal synapses. This could be achieved using the human synapsin (hSYN) promoter, which was shown to be a first-class promoter in driving transgene expression in corticospinal neurons, as it is neuron-specific and drives strong expression [57]. Furthermore, the use of different AAV capsids, such as RH10 or AAV9-PhP.B, may also largely improve the targeting of neurons following systemic delivery or direct brain administration [58,59,60,61]. One should also consider cloning the Dp71f sequence under the control of the hSYN promoter to restore expression of this synaptic Dp71 isoform. Ideally, a cotreatment should be considered to restore both Dp71d in glial cells and Dp71f in synapses. Finally, other behavioral outcome measures could be tested, as Dp71-null mice also exhibit altered spatial working memory and cognitive flexibility [22,23], which are critical processes for intellectual functioning in humans [1,62].

## 5. Conclusions

We demonstrated the potential, as well as the limitations, of AAV-mediated restoration of Dp71 expression in the brain of Dp71-null mice following early postnatal ICP4 delivery (P4) and ICV delivery in adults. Although we achieved substantial rescue of Dp71 expression, mainly in glial-cell endfeet, and even overexpression in the hippocampus and cortex following ICV delivery, this was, however, not sufficient to compensate for the altered emotional behavior displayed by this mouse model. Nevertheless, we showed that Dp71 rescue was associated with a localization of key associated proteins, α1-syntrophin and AQP4 water channels, thus demonstrating that detection of these proteins can be considered as relevant cellular markers of Dp71 rescue. Our results suggest that this type of gene therapy has the potential to restore this key membrane scaffold of proteins normally involved in blood–brain barrier functions and water homeostasis. Alternative AAV serotypes, promoters and Dp71 isoform sequences should be considered in future studies to drive higher levels of expression and/or specific expression of additional synaptic isoforms of Dp71. Such a therapeutic approach is particularly relevant for patients with distal mutations in the *DMD* gene affecting expression of Dp71. Indeed, for the most part, these DMD patients are not eligible for exon-skipping treatments that would alter key functional domains of the protein. Our encouraging results provide hope for the future development of gene therapies amenable to the restoration of brain comorbidities associated with Dp71 loss of function, which would constitute a huge step forward toward improving the lifespan conditions of a severely affected subgroup of DMD patients.

## Data Availability

The primary data for this study are available from the authors upon request.

## Supplementary Materials

The following supporting information can be downloaded at: https://www.mdpi.com/article/10.3390/cells13080718/s1, Figure S1: Additional parameters analyzed in the behavioral study. (A–C) The plots show the data recorded following intracardiac injections at P4 (ICP4) in the three groups of mice (Dp71-T, n = 7; WT-C, n = 9; Dp71-C, n = 7). (A) Number of hesitations and rearings in the light–dark choice test (LD). (B) Number of entries in the center and in open arms expressed as % of total number of visits in all zones of the maze, and time spent in the center and in open arms (s) in the elevated plus maze (EPM). (C) Maximum speed and time spent in the center (s) in the 30 min open-field exploration test (OF). (D–F) The plots show the data recorded following stereotaxic intracerebroventricular (ICV) injections at 6-8 weeks in the three groups of mice (Dp71-T, n = 6; WT-C, n = 6; Dp71-C, n = 6). (D) Number of hesitations and rearings in the light–dark choice test (LD). (E) Number of entries in the center and in open arms expressed as % of total number of visits in all zones of the maze, and time spent in the center and in open arms (s) in the elevated plus maze (EPM). (F) Maximum speed and time spent in the center (s) in the 30 min open-field exploration test (OF). The results are the means ± SEM, * *p* < 0.05, Mann–Whitney test.

## References

[1] Ricotti V., Mandy W.P., Scoto M., Pane M., Deconinck N., Messina S., Mercuri E., Skuse D.H., Muntoni F. (2016). Neurodevelopmental, emotional, and behavioural problems in Duchenne muscular dystrophy in relation to underlying dystrophin gene mutations. Dev. Med. Child Neurol..

[2] Waite A., Tinsley C.L., Locke M., Blake D.J. (2009). The neurobiology of the dystrophin-associated glycoprotein complex. Ann. Med..

[3] Desguerre I., Christov C., Mayer M., Zeller R., Becane H.M., Bastuji-Garin S., Leturcq F., Chiron C., Chelly J., Gherardi R.K. (2009). Clinical heterogeneity of duchenne muscular dystrophy (DMD): Definition of sub-phenotypes and predictive criteria by long-term follow-up. PLoS ONE.

[4] Daoud F., Angeard N., Demerre B., Martie I., Benyaou R., Leturcq F., Cossee M., Deburgrave N., Saillour Y., Tuffery S. (2009). Analysis of Dp71 contribution in the severity of mental retardation through comparison of Duchenne and Becker patients differing by mutation consequences on Dp71 expression. Hum. Mol. Genet..

[5] Daoud F., Candelario-Martinez A., Billard J.M., Avital A., Khelfaoui M., Rozenvald Y., Guegan M., Mornet D., Jaillard D., Nudel U. (2009). Role of mental retardation-associated dystrophin-gene product Dp71 in excitatory synapse organization, synaptic plasticity and behavioral functions. PLoS ONE.

[6] Haenggi T., Soontornmalai A., Schaub M.C., Fritschy J.M. (2004). The role of utrophin and Dp71 for assembly of different dystrophin-associated protein complexes (DPCs) in the choroid plexus and microvasculature of the brain. Neuroscience.

[7] Nicchia G.P., Rossi A., Nudel U., Svelto M., Frigeri A. (2008). Dystrophin-dependent and -independent AQP4 pools are expressed in the mouse brain. Glia.

[8] Belmaati Cherkaoui M., Vacca O., Izabelle C., Boulay A.C., Boulogne C., Gillet C., Barnier J.V., Rendon A., Cohen-Salmon M., Vaillend C. (2021). Dp71 contribution to the molecular scaffold anchoring aquaporine-4 channels in brain macroglial cells. Glia.

[9] Vacca O., Charles-Messance H., El Mathari B., Sene A., Barbe P., Fouquet S., Aragon J., Darche M., Giocanti-Auregan A., Paques M. (2016). AAV-mediated gene therapy in Dystrophin-Dp71 deficient mouse leads to blood-retinal barrier restoration and oedema reabsorption. Hum Mol Genet.

[10] Naidoo M., Anthony K. (2020). Dystrophin Dp71 and the Neuropathophysiology of Duchenne Muscular Dystrophy. Mol. Neurobiol..

[11] Wijekoon N., Gonawala L., Ratnayake P., Amaratunga D., Hathout Y., Mohan C., Steinbusch H.W.M., Dalal A., Hoffman E.P., de Silva K.R.D. (2023). Duchenne Muscular Dystrophy from Brain to Muscle: The Role of Brain Dystrophin Isoforms in Motor Functions. J. Clin. Med..

[12] Connors N.C., Adams M.E., Froehner S.C., Kofuji P. (2004). The potassium channel Kir4.1 associates with the dystrophin-glycoprotein complex via alpha-syntrophin in glia. J. Biol. Chem..

[13] Galaz-Vega R., Hernandez-Kelly L.C., Mendez J.A., Cisneros B., Ortega A. (2005). Glutamate regulates dystrophin-71 levels in glia cells. Neurochem. Res..

[14] Fort P.E., Sene A., Pannicke T., Roux M.J., Forster V., Mornet D., Nudel U., Yaffe D., Reichenbach A., Sahel J.A. (2008). Kir4.1 and AQP4 associate with Dp71- and utrophin-DAPs complexes in specific and defined microdomains of Muller retinal glial cell membrane. Glia.

[15] Fujimoto T., Stam K., Yaoi T., Nakano K., Arai T., Okamura T., Itoh K. (2023). Dystrophin Short Product, Dp71, Interacts with AQP4 and Kir4.1 Channels in the Mouse Cerebellar Glial Cells in Contrast to Dp427 at Inhibitory Postsynapses in the Purkinje Neurons. Mol. Neurobiol..

[16] Nicchia G.P., Nico B., Camassa L.M., Mola M.G., Loh N., Dermietzel R., Spray D.C., Svelto M., Frigeri A. (2004). The role of aquaporin-4 in the blood-brain barrier development and integrity: Studies in animal and cell culture models. Neuroscience.

[17] Vajda Z., Pedersen M., Fuchtbauer E.M., Wertz K., Stodkilde-Jorgensen H., Sulyok E., Doczi T., Neely J.D., Agre P., Frokiaer J. (2002). Delayed onset of brain edema and mislocalization of aquaporin-4 in dystrophin-null transgenic mice. Proc. Natl. Acad. Sci. USA.

[18] Amiry-Moghaddam M., Frydenlund D.S., Ottersen O.P. (2004). Anchoring of aquaporin-4 in brain: Molecular mechanisms and implications for the physiology and pathophysiology of water transport. Neuroscience.

[19] Benabdesselam R., Sene A., Raison D., Benmessaoud-Mesbah O., Ayad G., Mornet D., Yaffe D., Rendon A., Hardin-Pouzet H., Dorbani-Mamine L. (2010). A deficit of brain dystrophin 71 impairs hypothalamic osmostat. J. Neurosci. Res..

[20] Lange J., Gillham O., Alkharji R., Eaton S., Ferrari G., Madej M., Flower M., Tedesco F.S., Muntoni F., Ferretti P. (2022). Dystrophin deficiency affects human astrocyte properties and response to damage. Glia.

[21] Vaillend C., Ungerer A. (1999). Behavioral characterization of *mdx3cv* mice deficient in C-terminal dystrophins. Neuromuscul. Disord..

[22] Helleringer R., Le Verger D., Li X., Izabelle C., Chaussenot R., Belmaati-Cherkaoui M., Dammak R., Decottignies P., Daniel H., Galante M. (2018). Cerebellar synapse properties and cerebellum-dependent motor and non-motor performance in Dp71-null mice. Dis. Model. Mech..

[23] Chaussenot R., Amar M., Fossier P., Vaillend C. (2019). Dp71-Dystrophin Deficiency Alters Prefrontal Cortex Excitation-Inhibition Balance and Executive Functions. Mol. Neurobiol..

[24] Hammond S.M., Aartsma-Rus A., Alves S., Borgos S.E., Buijsen R.A.M., Collin R.W.J., Covello G., Denti M.A., Desviat L.R., Echevarria L. (2021). Delivery of oligonucleotide-based therapeutics: Challenges and opportunities. EMBO Mol. Med..

[25] Filonova G., Aartsma-Rus A. (2023). Next steps for the optimization of exon therapy for Duchenne muscular dystrophy. Expert Opin. Biol. Ther..

[26] Saoudi A., Barberat S., le Coz O., Vacca O., Doisy Caquant M., Tensorer T., Sliwinski E., Garcia L., Muntoni F., Vaillend C. (2023). Partial restoration of brain dystrophin by tricyclo-DNA antisense oligonucleotides alleviates emotional deficits in *mdx52* mice. Mol. Ther. Nucleic Acids..

[27] Zarrouki F., Relizani K., Bizot F., Tensorer T., Garcia L., Vaillend C., Goyenvalle A. (2022). Partial Restoration of Brain Dystrophin and Behavioral Deficits by Exon Skipping in the Muscular Dystrophy X-Linked (*mdx*) Mouse. Ann. Neurol..

[28] Sekiguchi M., Zushida K., Yoshida M., Maekawa M., Kamichi S., Yoshida M., Sahara Y., Yuasa S., Takeda S., Wada K. (2009). A deficit of brain dystrophin impairs specific amygdala GABAergic transmission and enhances defensive behaviour in mice. Brain.

[29] Vacca O., Darche M., Schaffer D.V., Flannery J.G., Sahel J.A., Rendon A., Dalkara D. (2014). AAV-mediated gene delivery in Dp71-null mouse model with compromised barriers. Glia.

[30] Barboni M.T.S., Vaillend C., Joachimsthaler A., Liber A.M.P., Khabou H., Roux M.J., Vacca O., Vignaud L., Dalkara D., Guillonneau X. (2020). Rescue of Defective Electroretinographic Responses in Dp71-Null Mice With AAV-Mediated Reexpression of Dp71. Investig. Ophthalmol. Vis. Sci..

[31] Cearley C.N., Wolfe J.H. (2006). Transduction characteristics of adeno-associated virus vectors expressing cap serotypes 7, 8, 9, and Rh10 in the mouse brain. Mol. Ther..

[32] Muramatsu K., Muramatsu S.I. (2023). Adeno-associated virus vector-based gene therapies for pediatric diseases. Pediatr. Neonatol..

[33] Sarig R., Mezger-Lallemand V., Gitelman I., Davis C., Fuchs O., Yaffe D., Nudel U. (1999). Targeted inactivation of Dp71, the major non-muscle product of the *DMD* gene: Differential activity of the Dp71 promoter during development. Hum. Mol. Genet..

[34] Xu L., Daly T., Gao C., Flotte T.R., Song S., Byrne B.J., Sands M.S., Parker Ponder K. (2001). CMV-beta-actin promoter directs higher expression from an adeno-associated viral vector in the liver than the cytomegalovirus or elongation factor 1 alpha promoter and results in therapeutic levels of human factor X in mice. Hum. Gene. Ther..

[35] Niwa H., Yamamura K., Miyazaki J. (1991). Efficient selection for high-expression transfectants with a novel eukaryotic vector. Gene.

[36] Choi V.W., Asokan A., Haberman R.A., Samulski R.J. (2007). Production of recombinant adeno-associated viral vectors. Curr. Protoc. Hum. Genet..

[37] Aurnhammer C., Haase M., Muether N., Hausl M., Rauschhuber C., Huber I., Nitschko H., Busch U., Sing A., Ehrhardt A. (2012). Universal real-time PCR for the detection and quantification of adeno-associated virus serotype 2-derived inverted terminal repeat sequences. Hum. Gene Ther. Methods.

[38] Paxinos G., Franklin K.B.J. (2001). The Mouse Brain in Stereotaxic Coordinates.

[39] Zar J.H. (1984). Biostatistical Analysis.

[40] Zincarelli C., Soltys S., Rengo G., Rabinowitz J.E. (2008). Analysis of AAV serotypes 1-9 mediated gene expression and tropism in mice after systemic injection. Mol. Ther..

[41] Foust K.D., Nurre E., Montgomery C.L., Hernandez A., Chan C.M., Kaspar B.K. (2009). Intravascular AAV9 preferentially targets neonatal neurons and adult astrocytes. Nat. Biotechnol..

[42] Lowenstein P.R. (2009). Crossing the rubicon. Nat. Biotechnol..

[43] Saunders N.R., Joakim Ek C., Dziegielewska K.M. (2009). The neonatal blood-brain barrier is functionally effective, and immaturity does not explain differential targeting of AAV9. Nat. Biotechnol..

[44] Reemst K., Noctor S.C., Lucassen P.J., Hol E.M. (2016). The Indispensable Roles of Microglia and Astrocytes during Brain Development. Front. Hum. Neurosci..

[45] Rincon M.Y., de Vin F., Duque S.I., Fripont S., Castaldo S.A., Bouhuijzen-Wenger J., Holt M.G. (2018). Widespread transduction of astrocytes and neurons in the mouse central nervous system after systemic delivery of a self-complementary AAV-PHP.B vector. Gene Ther..

[46] McCarty D.M. (2008). Self-complementary AAV vectors; advances and applications. Mol. Ther..

[47] Lee S.H., Yang J.Y., Madrakhimov S., Park H.Y., Park K., Park T.K. (2019). Adeno-Associated Viral Vector 2 and 9 Transduction Is Enhanced in Streptozotocin-Induced Diabetic Mouse Retina. Mol. Ther. Methods Clin. Dev..

[48] Saoudi A., Zarrouki F., Sebrie C., Izabelle C., Goyenvalle A., Vaillend C. (2021). Emotional behavior and brain anatomy of the *mdx52* mouse model of Duchenne muscular dystrophy. Dis. Model. Mech..

[49] Maresh K., Papageorgiou A., Ridout D., Harrison N.A., Mandy W., Skuse D., Muntoni F. (2023). Startle responses in Duchenne muscular dystrophy: A novel biomarker of brain dystrophin deficiency. Brain.

[50] Alemdaroglu-Gurbuz I., Ipek C., Bulut N., Karaduman A., Yilmaz O. (2022). The Impact of “Fear of Falling” on Physical Performance, Balance, and Ambulation in Duchenne Muscular Dystrophy. Neuropediatrics.

[51] Austin R.C., Morris G.E., Howard P.L., Klamut H.J., Ray P.N. (2000). Expression and synthesis of alternatively spliced variants of Dp71 in adult human brain. Neuromuscul. Disord..

[52] Tillfors M., Furmark T., Marteinsdottir I., Fredrikson M. (2002). Cerebral blood flow during anticipation of public speaking in social phobia: A PET study. Biol. Psychiatry.

[53] Evans K.C., Wright C.I., Wedig M.M., Gold A.L., Pollack M.H., Rauch S.L. (2008). A functional MRI study of amygdala responses to angry schematic faces in social anxiety disorder. Depress. Anxiety.

[54] Sakai Y., Kumano H., Nishikawa M., Sakano Y., Kaiya H., Imabayashi E., Ohnishi T., Matsuda H., Yasuda A., Sato A. (2005). Cerebral glucose metabolism associated with a fear network in panic disorder. Neuroreport.

[55] Wang T., Liu J., Zhang J., Zhan W., Li L., Wu M., Huang H., Zhu H., Kemp G.J., Gong Q. (2016). Altered resting-state functional activity in posttraumatic stress disorder: A quantitative meta-analysis. Sci. Rep..

[56] Chao O.Y., Pathak S.S., Zhang H., Augustine G.J., Christie J.M., Kikuchi C., Taniguchi H., Yang Y.M. (2023). Social memory deficit caused by dysregulation of the cerebellar vermis. Nat. Commun..

[57] Nieuwenhuis B., Haenzi B., Hilton S., Carnicer-Lombarte A., Hobo B., Verhaagen J., Fawcett J.W. (2021). Optimization of adeno-associated viral vector-mediated transduction of the corticospinal tract: Comparison of four promoters. Gene Ther..

[58] Klein R.L., Dayton R.D., Tatom J.B., Henderson K.M., Henning P.P. (2008). AAV8, 9, Rh10, Rh43 vector gene transfer in the rat brain: Effects of serotype, promoter and purification method. Mol. Ther..

[59] Hu C., Busuttil R.W., Lipshutz G.S. (2010). RH10 provides superior transgene expression in mice when compared with natural AAV serotypes for neonatal gene therapy. J. Gene Med..

[60] Pietersz K.L., Plessis F.D., Pouw S.M., Liefhebber J.M., van Deventer S.J., Martens G.J.M., Konstantinova P.S., Blits B. (2021). PhP.B Enhanced Adeno-Associated Virus Mediated-Expression Following Systemic Delivery or Direct Brain Administration. Front. Bioeng. Biotechnol..

[61] Surdyka M., Jesion E., Niewiadomska-Cimicka A., Trottier Y., Kalinowska-Poska Z., Figiel M. (2022). Selective transduction of cerebellar Purkinje and granule neurons using delivery of AAV-PHP.eB and AAVrh10 vectors at axonal terminal locations. Front. Mol. Neurosci..

[62] de Brouwer A.P., Nabuurs S.B., Verhaart I.E., Oudakker A.R., Hordijk R., Yntema H.G., Hordijk-Hos J.M., Voesenek K., de Vries B.B., van Essen T. (2014). A 3-base pair deletion, c.9711_9713del, in DMD results in intellectual disability without muscular dystrophy. Eur. J. Hum. Genet..

