# Supplementary material for: AAV-Mediated Restoration of Dystrophin-Dp71 in the Brain of Dp71-Null Mice: Molecular, Cellular and Behavioral Outcomes
Source: Cells. 2024 Apr 20;13(8):718. doi: 10.3390/cells13080718 (PMC11049308; doi:10.3390/cells13080718)
Supplement: Supplementary file 1 [file cells-13-00718-s001.zip › cells-2918873-supplementary.pdf]

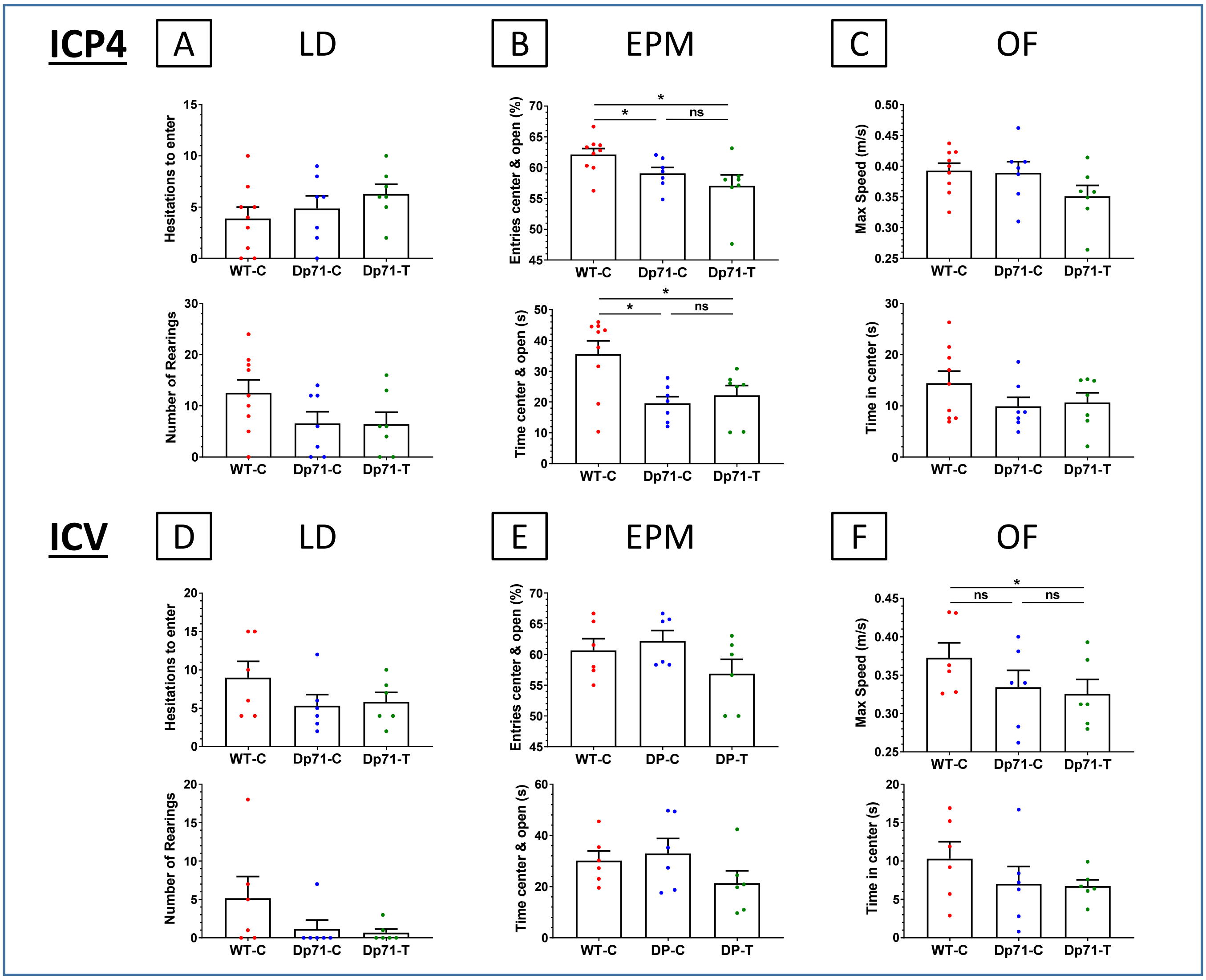

Figure S1: Additional parameters analyzed in the behavioral study. (**A-C**) The plots show the data recorded following intracardiac injections at P4 (IC-P4) in the three groups of mice (Dp71-T, n=7; WT-C, n=9; Dp71-C, n=7). (**A**) Number of hesitations and rearings in the light/dark choice test (LD). (**B**) Number of entries in the center and in open arms expressed as % of total number of visits in all zones of the maze, and time spent in the center and in open arms (s) in the elevated plus maze (EPM). (**C**) Maximum speed and time spent in the center (s) in the 30-min open-field exploration test (OF). (**D-F**) The plots show the data recorded following stereotaxic intracerebroventricular (ICV) injections at 6-8 weeks in the three groups of mice (Dp71-T, n=6; WT-C, n=6; Dp71-C, n=6). (**D**) Number of hesitations and rearings in the light/dark choice test (LD). (**E**) Number of entries in the center and in open arms expressed as % of total number of visits in all zones of the maze, and time spent in the center and in open arms (s) in the elevated plus maze (EPM). (**F**) Maximum speed and time spent in the center (s) in the 30-min open-field exploration test (OF). Results are means ± SEM, *p<0.05, Mann-Whitney test.
